# Exploring the possible relationship between skin microbiome and brain cognitive functions: a pilot EEG study

**DOI:** 10.1038/s41598-024-57649-z

**Published:** 2024-04-02

**Authors:** Po-Chun Wang, Daniyal Rajput, Xin-Fu Wang, Chun-Ming Huang, Chun-Chuan Chen

**Affiliations:** 1 Department of Biomedical Sciences and Engineering, National Central University, Taoyuan, Taiwan; 2 Taiwan International Graduate Program in Interdisciplinary Neuroscience, National Central University and Academia Sinica, Taipei, Taiwan; 3 Institute of Cognitive Neuroscience, National Central University, Taoyuan, Taiwan

**Keywords:** Microbiology, Neuroscience, Physiology, Neurology

## Abstract

Human microbiota mainly resides on the skin and in the gut. Human gut microbiota can produce a variety of short chain fatty acids (SCFAs) that affect many physiological functions and most importantly modulate brain functions through the bidirectional gut-brain axis. Similarly, skin microorganisms also have identical metabolites of SCFAs reported to be involved in maintaining skin homeostasis. However, it remains unclear whether these SCFAs produced by skin bacteria can affect brain cognitive functions. In this study, we hypothesize that the brain’s functional activities are associated with the skin bacterial population and examine the influence of local skin-bacterial growth on event-related potentials (ERPs) during an oddball task using EEG. Additionally, five machine learning (ML) methods were employed to discern the relationship between skin microbiota and cognitive functions. Twenty healthy subjects underwent three rounds of tests under different conditions—alcohol, glycerol, and water. Statistical tests confirmed a significant increase in bacterial population under water and glycerol conditions when compared to the alcohol condition. The metabolites of bacteria can turn phenol red from red–orange to yellow, confirming an increase in acidity. P3 amplitudes were significantly enhanced in response to only oddball stimulus at four channels (Fz, FCz, and Cz) and were observed after the removal of bacteria when compared with that under the water and glycerol manipulations. By using machine learning methods, we demonstrated that EEG features could be separated with a good accuracy (> 88%) after experimental manipulations. Our results suggest a relationship between skin microbiota and brain functions. We hope our findings motivate further study into the underlying mechanism. Ultimately, an understanding of the relationship between skin microbiota and brain functions can contribute to the treatment and intervention of diseases that link with this pathway.

## Introduction

Human microbiota is a set of large heterogeneous microorganisms which largely reside on the skin and in the gut. The existence of human microbiota is beneficial, pathogenic, or mutualistic. Previous studies reported that gut microbiota produces short chain fatty acids (SCFAs) to affect the host, such as the homeostasis of intestines, the energy balance, modulating mucosal homeostasis of immune cell subpopulations and the synthesis of certain vitamins^1–4^. Furthermore, it was reported that gut microbiota may play a crucial role in cross-talking between the gut and the brain through bidirectional signaling mechanisms, known as the gut-brain axis (GBA). These signaling mechanisms run between the gastrointestinal tract and the central nervous system, although the definite mechanism is still inconclusive^4–6^. For instance, the brain can modulate the secretory and sensorimotor functions of the gut, such as controlling gut hormones and the gastrointestinal barrier^4,6^. Importantly, the diversity of the gastrointestinal bacterial population significantly affects cognition. Li et al., observed an increasing in working and long-term memory in mice with a specific diet (ground beef chow) than with standard diet, owing to a notable increase in bacterial diversity^7^. Similarly, the consumption of probiotics improves the attention to positive stimuli and modulates emotions (see^8,9^ for a review). In contrast, bacteria’s imbalance in intestinal composition was associated with various disorders, such as autism spectrum disorders (ASD), depression, stress, and communication^10^. Adams, et al. demonstrated that the faeces of ASD children who did not receive probiotics had significantly lower SCFAs (acetate, propionate, and valerate) and lower Bifidobacter species and higher Lactobacillus species when compared to healthy children and ASD children who received probiotics^11^. Likewise, Parkinson’s patients exhibited a lower concentration of SCFA in faeces due to lower SCFAs producing gut bacteria than healthy controls^12^. These findings indicate that gut microbiota plays an essential role in regulating brain functions and a variety of SCFAs are involved in mediating communications between gut and brain.

Like the gut, microorganisms on the skin play a critical role in maintaining skin homeostasis, protecting against pathogens and harmful chemicals, regulating our immune system, and decomposing natural products^13–15^. Bacterial growth on human skin is influenced by skin microenvironments, age, sebum level, hormonal level, and the production of sweat^13,16^. Imbalances in skin microbiota (also called dysbiosis) can cause several cutaneous diseases, such as atopic dermatitis, wounds, psoriasis, acne vulgaris, diabetic foot ulcer, or Pityriasis Versicolor^14,15^. It has been reported that Staphylococcus epidermidis can produce SCFA through fermentation to inhibit the growth of Cutibacterium acnes, the cause of acne inflammation^17^ or to attenuate UV-irradiated skin inflammation by modulating FFAR2 or HDAC and activating G protein-coupled receptors^18,19^. In 2021, we found that the SCFA produced by Cutibacterium acnes can pass through the skin and participate in the inhibition of melanin production without causing cell damage and disrupting the balance of the skin microbiome in vivo^20^. In short, skin microbiomes are essential for sustaining skin homeostasis and health. Given that the metabolites of skin bacteria were also SCFAs, it is suspected that the skin microbiome can affect cognitive functions.

Electroencephalography (EEG) measures electrical activity of the brain and has been widely used for detecting brain abnormalities given a specific task or condition. For instance, the oddball paradigm is one of the most-used tasks to investigate cognitive processes of attention. Specifically, the event-related potentials (ERPs) of N200 (a negative peak around 200 ms) and P300 (a positive peak around 300 ms) after stimulus were used to indicate higher-level cognitive processes like selective attention and memory updating^21,22^. Previous studies have suggested that the enhanced N200 amplitudes are related to the selective allocation of spatial attention^23^, the detection of novelty or mismatch^24^ and cognitive control^25^, while the enhanced P300 amplitudes are related to selective attention^26^. Abnormalities in the amplitudes or latencies of N200 and P300 may also be related to diseases such as attention deficit hyperactivity disorder^27^. Hence, the above findings suggest that an oddball task is an efficient way to measure human attention by examining the ERP components.

In summary, prior studies have investigated the gut-brain axis and identified the functional roles of SCFA; however, it remains unclear whether the skin microbiome of the same metabolites affects cognitive functions. To test this, we designed this study to establish the interaction between the skin microbiome and brain signals related to cognitive functions of attention. First, we manipulated the bacterial population on the forehead of healthy participants. Then, we recorded the EEG of each participant during an oddball task and examined their ERPs’ components (N2 and P3). Finally, we applied statistical and machine learning (ML) methods to discern the association between the skin microbiota and cognitive function.

## Methodology

### Subjects and task

Twenty-four healthy participants (20–35 years of age) were recruited for this study, but four subjects were excluded due to their failure to follow given instructions during an oddball task, resulting in 14 males and 6 females in this study (mean age: 25.5 ± 3.2 years). None reported a personal history of psychiatric or neurological disease or substance abuse. We measured the attention of each participant using a classical oddball paradigm^28,29^. The task comprised two auditory stimuli (odd and standard) with different frequencies, and these stimuli were presented in a random series. Odd-tone (or target tone) stimuli had higher frequency (2000 Hz) and were presented infrequently (100 ± 10 trail, 20% occurrence rate), whereas standard-tone had lower frequency (1000 Hz) and frequently occurred (400 ± 40 trail, 80% occurrence rate). All subjects were instructed to mentally count the number of odd tones as quickly and correctly as possible while ignoring the standard tones. The odd-tone stimuli were designed to elicit the P300 due to higher concentration than standard stimuli. Each participant completed the oddball task three times with different experimental manipulations of bacteria (see also “Bacterial manipulation and measurement” for details) in a randomized order. There was always a one-week interval between the participant’s latest experiments to minimize the impact of memorization and habituation effects.

### Ethical approval

Experimental procedures were designed in accordance with the Declaration of Helsinki and were approved by the Research Ethics Committee of China Medical University & Hospital (approval No. CMUH107-REC3-152). Written informed consent was obtained from all participants for the experiments.

### Bacterial manipulation and measurement

To measure the influence of bacteria on cognition, we designed three experiments to manipulate the bacterial population on the forehead of each participant and then recorded brain activity by employing an oddball paradigm. The three experiments are alcohol, glycerol, and water manipulations. To mitigate potential confounding effects of order, the three experiments were randomized using the simple randomization method^30^. Due to the capability of *Cutibacterium acnes*^20^ and *S. epidermidis*^17^ to produce SCFAs and their abundance on facial skin, we selected the forehead for all experimental manipulations. The decision to avoid the cheeks was rooted in concerns related to the experimental manipulation of alcohol. Alcohol is volatile and pungent, and excessive use on the skin could be irritating. Specifically, for the alcohol manipulation, we applied 75% alcohol to eliminate bacteria 5 min before conducting an oddball task. For the water manipulation, we applied double distilled water on the forehead to mimic natural skin microbial growth and then waited for 5 min before initiating an oddball task. For the glycerol manipulation, 65% glycerol was applied to the forehead to increase the bacterial population. Glycerol is a major source of carbon and bacteria use it as a nutrient to grow. Following the application of glycerol, we covered the subject’s forehead with plastic wrap for 6 h to foster bacterial growth while minimizing the risk of bacterial contamination. An initial pilot study was conducted to establish the growth curve of forehead bacteria in the presence of glycerol. Based on the growth curve analysis, we determined that a 6-h period represents the optimal timing for extracting the bacterial population given the glycerol concentration.

To determine the bacterial growth, skin samples from all experiments were incubated in 10 mL rich media (3 g/L TSB) in the presence of C12-14 alkyl benzoate, Cetyl ethylhexanoate, and Cetearyl isononanoate under aerobic conditions at 37 °C with shaking at 200 rpm for 2 days. This media was first mixed with the 0.002% (w/v) phenol red (Sigma), a pH indicator, to measure the acidity. A color change from red–orange to yellow indicated an increase in acidity resulting from bacterial fermentation. Finally, the bacterial population was quantified by the measurement of the optical density at OD562 nm.

### EEG acquisition and processing

Fourteen channel EEG (10–20 system; C3, C4, Cz, F3, F4, FC1, FC2, Fz, FP1, FP2, FCz, Pz, T3, and T4) were recorded during the oddball tasks of three different experiments (alcohol, water, and glycerol) at a sampling rate of 250 Hz. Two electrooculograms (EOG), recorded from horizontal and vertical electrodes positioned around the right eye, were also recorded throughout the experiment. For pre-processing, the EEG data were first band-pass filtered (0.1–30 Hz) using fifth-order Butterworth filter and epoched with a peristimulus window (from 500 to 1000 ms), where time zero denotes the initiation of the auditory stimulus, offline using SPM12 (Wellcome Trust Centre for Neuroimaging, http://www.fil.ion.ucl.ac.uk/spm/). Then, a fully automated correction method was applied to remove EOG contamination across all trials^31^. Basically, this method is based on a regression model to eliminate fast and slow EOG-related artifacts. In this regression model, the measured EOG data were treated as independent noise to EEG, assuming a zero inner product between noise (i.e. EOG) and signals (i.e. EEG). This enables the estimation of weighting coefficients for EOG contamination in EEG. Subsequently, the EOG components in EEG can be removed by projecting them out using the estimated weighting coefficients, effectively minimizing the influence of EOG interferences in EEG signals. This method is selected for its simplicity and robustness, and it can be applied with any number of EEG channels as long as EOG signals are available (refer to Ref.^31^ for further details). The EOG removed trials were divided into standard and odd stimuli. For ERPs analysis, trials were averaged across standard and odd trials and a baseline was corrected between − 150 and − 100 ms. The time windows for N2 and P3 were from 150 to 400 and from 300 to 700 ms after auditory cue, respectively. We extracted four ERPs parameters for comparison: N2 and P3 peak amplitudes and peak latencies.

### Statistical analysis

The OD values obtained before and after three experimental manipulations, including water, alcohol, and glycerol, that reflected the population of bacteria were entered into the repeated-measures analysis of variance (rmANOVA) with two factors of phase (2 levels: before and after) and manipulation (3 levels: water, alcohol, and glycerol). Similarly, the peak amplitude and latency of N2 and P3 were statistically tested by rmANOVA with experimental manipulations (alcohol, water, and glycerol) and conditions (standard and odd stimuli) at all channels. Prior to conducting the rmANOVA, both OD values and EEG features were assessed for normality using the Shapiro–Wilk normality test. This test was performed using MATLAB open source code obtained from the MATLAB Central File Exchange (https://www.mathworks.com/matlabcentral/fileexchange/13964-shapiro-wilk-and-shapiro-francia-normality-tests). A post hoc t-test was applied to further identify the significant pairwise differences among the factors. The reported results were considered statistically significant when p < 0.05 after correction for multiple comparisons using the Tukey’s test for pairwise mean comparisons.

### Machine learning and features

To examine the impacts of experimental manipulations on EEG changes, we further employed machine learning methods for multivariate analysis of EEG. Four well-known classifiers: support vector machine, Naïve Bayes, decision tree, and neural network, were used for 2-class classification to separate different datasets. We extracted only features demonstrating at least one statistically significant difference between experimental manipulations for each subject to alleviate overfitting bias. A ten-fold cross-validation method was used to evaluate the classification accuracy.

## Results

### Bacterial population under three experimental manipulations

**Figure 1:**
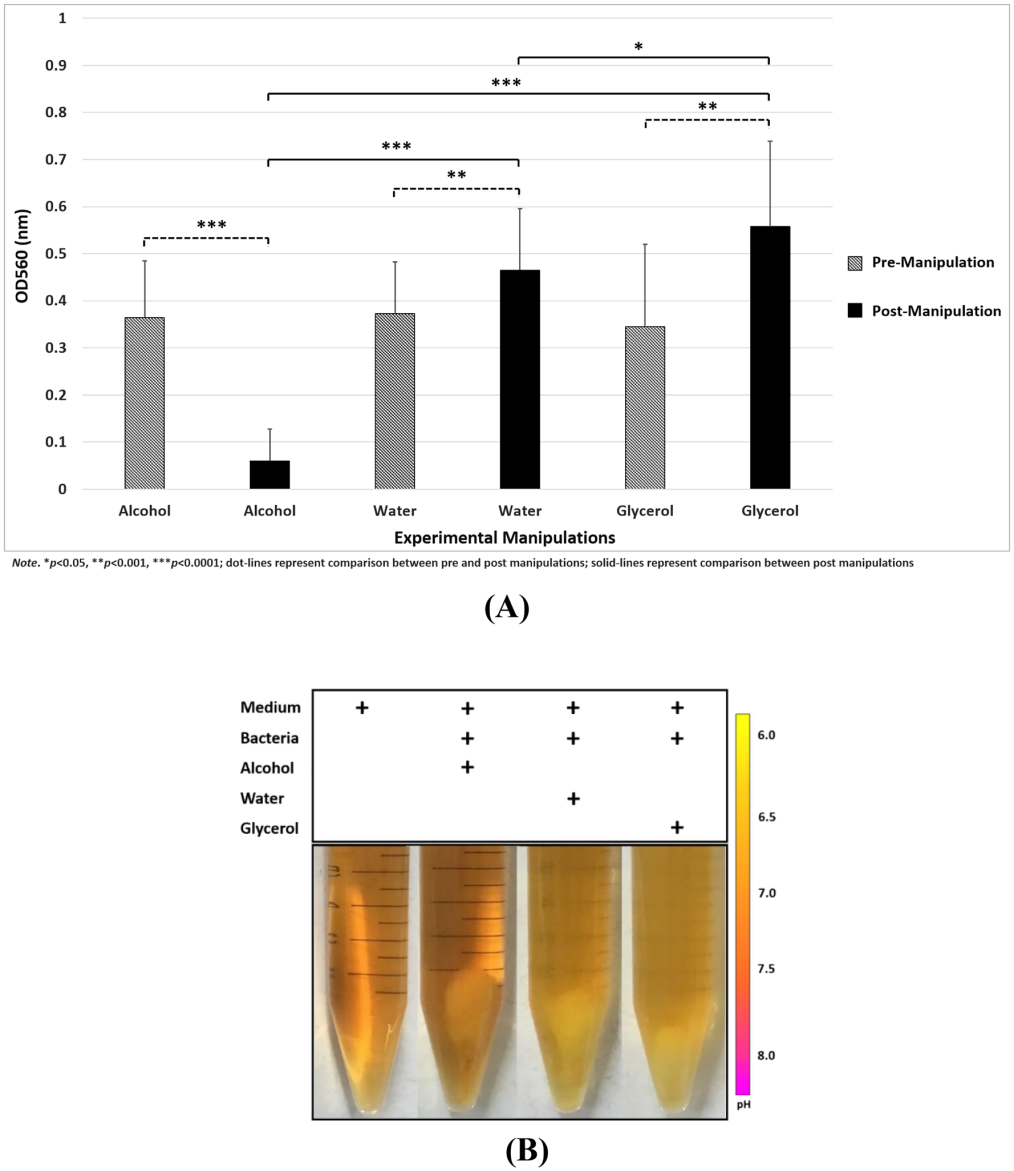
(**A**) Significant effects of experimental manipulations on bacteria population; (**B**) Color changes after experimental manipulations indicated bacterial fermentation.

We first examined the bacterial population measured with OD before and after applying alcohol, water, and glycerol. The rmANOVA exhibited a significant main factor of phase [F = 39.273, p < 0.0001]. Figure 1A depicted the significant differences in OD values before and after experimental manipulations [alcohol (t = 9.483, p < 0.0001); water (t = 4.349, p = 0.0003); glycerol (t = 4.177, p = 0.0005)] and between any two of the three experimental factors, including between glycerol and alcohol (t = − 9.483, p < 0.0001), between glycerol and water (t = − 2.527, p = 0.021), and between water and alcohol (t = − 11.117, p < 0.0001), identified by post hoc t-test. There were no significant differences between any pre-experimental measurements of OD values, suggesting a similar baseline condition. In addition, Fig. 1B depicted the experiment-specific color changes of phenol red due to bacterial fermentation. By visual inspection, it can be seen that the glycerol condition can result in lighter yellow, indicating a lower pH value and acidity of solution, whereas experiments with alcohol had the orange color of medium, suggesting relatively trivial changes in pH value. The water condition has a similar but not identical effect to that of the glycerol condition.

### ERPs results of the classical oddball task

**Figure 2:**
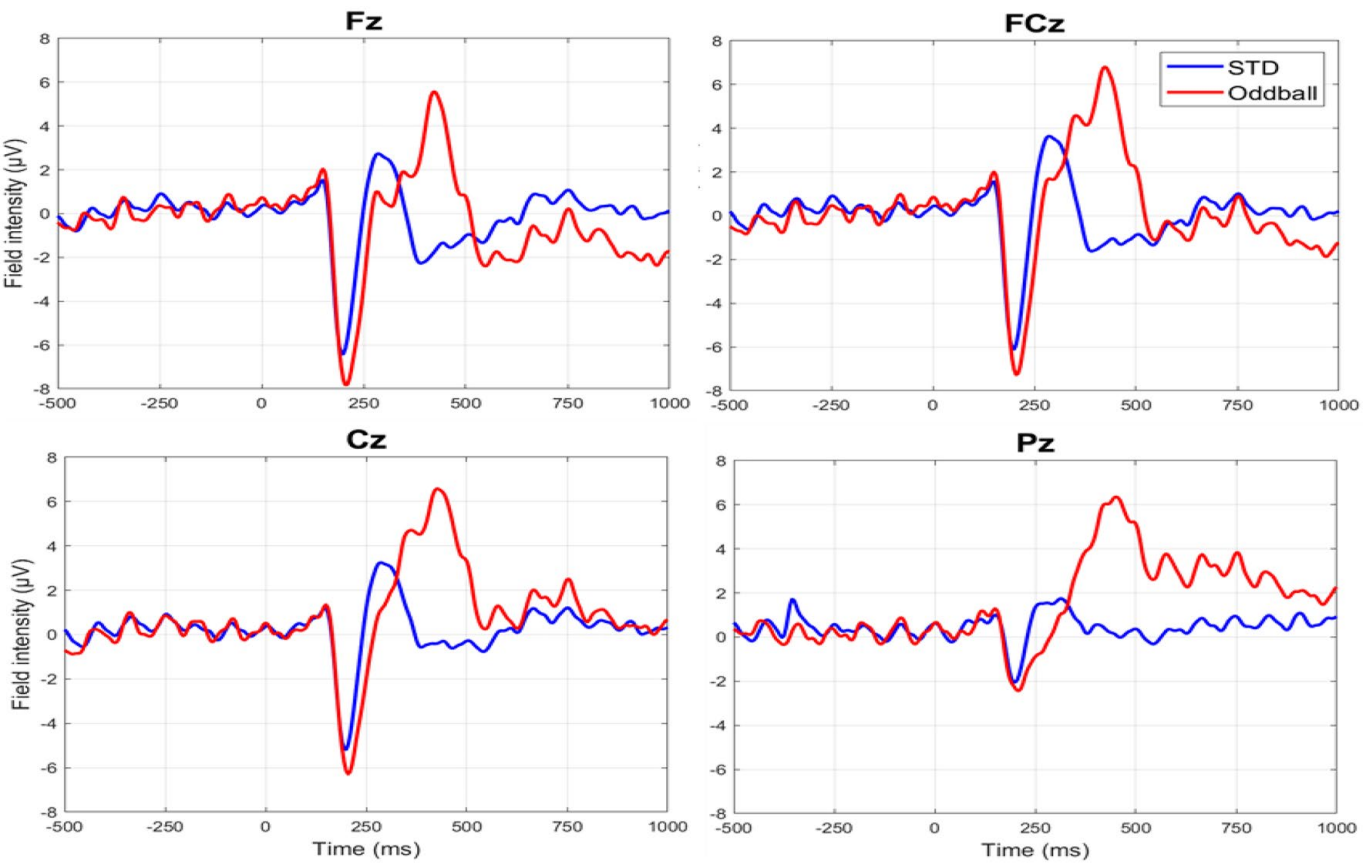
The time courses of ERPs during classical oddball task at Fz, FCz, Cz, and Pz.

To validate the efficacy of our experimental paradigm, we first examined the ERPs results of N2 and P3 during a classical oddball task under the water condition. Figure 2 shows the time courses of ERPs containing N2 and P3 at Fz, FCz, Cz, and Pz under the water condition. The rmANOVA confirmed a significant main effect of condition (oddball and standard trials) [F = 80, p < 0.0001] in peak amplitude. Post hoc paired t-tests confirmed significant differences in P3 peak at Fz, FCz, Cz and Pz, and in N2 peak at Fz and FCz (Table 1) between the oddball and standard trials. Similarly, the differences in latency between odd and standard trials were statistically significant at channels for Fz, FCz, Cz and Pz for both P3 and N2 components (Table 1).

**Table 1:** Statistical results of ERPs.

Channels	Paired t-test			
	Amplitude		Latency	
	P3	N2	P3	N2
Fz	< 0.0001	0.017	0.027	0.0001
FCz	< 0.0001	0.038	0.001	0.003
Cz	< 0.0001	0.079	< 0.0001	0.003
Pz	< 0.0001	0.102	0.033	0.004

### ERPs results of experimental factors

**Figure 3:**
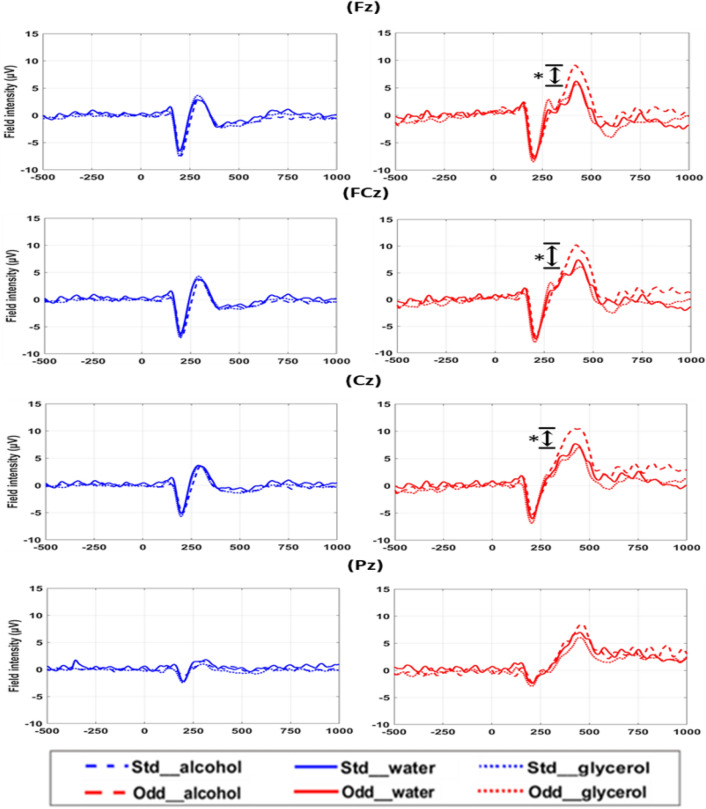
The time courses of ERPs three experimental factors.

To examine the experimental effects, the amplitudes and latency of N2 and P3 after applying water, alcohol and glycerol were compared. Figure 3 shows the time courses of ERPs with respect to the experimental effects and conditions. The rmANOVA indicated a significant main factor of experimental factor of amplitudes at Cz [F = 4.498, p = 0.0361] and FCz [F = 4.416, p = 0.0378] and a significant interaction between the experimental effect and conditions at Fz [F = 3.096, p = 0.0491] in P3 peak. The post hoc paired t-test revealed significant differences in P3 peak of oddball trials between alcohol and glycerol experiments at Fz (p = 0.0281), FCz (p = 0.0393) and Cz (p = 0.0495) (Fig. 3, marked with asterisks) after correction for multiple comparisons. Regarding N2, no significance was revealed by rmANOVA. Additionally, no significances in N2 or P3 were found in standard trials across three experimental factors and no significant changes were observed in latency.

### Machine learning methods differentiate the EEG data under different experimental manipulations

Finally, we applied machine learning methods to see whether the manipulations of skin bacteria alter the brain cognitive signals. In order to mitigate overfitting bias, we extracted features exclusively from Fz, FCz, Cz, and Pz with at least one statistically significant ERPs result, as outlined in Table 1. The features comprised a total of sixteen ERP features, encompassing both peak amplitudes and latencies, which were subsequently utilized in our machine learning analyses. Table 2 shows the classification accuracy using different classifiers. It can be seen that NN can best separate the EEG features between alcohol and water with 97.3% accuracy and between water and glycerol trials with an accuracy of 97.1%, while SVM can best distinguish the alcohol and glycerol trials. Importantly, the accuracy was always greater than 88%, irrelevant to experimental manipulations and classifiers.

**Table 2:** Machine learning classifiers to verify the influence of bacterial on EEG activity.

Class	SVM	NB	DT	NN
Alcohol vs glycerol	**95**	88.2	85.3	91.2
Alcohol vs water	97.1	96.5	88.2	**97.3**
Water vs glycerol	96.5	91.2	89.2	**97.1**

The highest accuracy among different classifiers for the given datasets is highlighted in bold.

## Discussion

In this study, we examined whether the presence of skin bacteria has any impact on EEG signals related to cognitive functions. We first examined the ERPs results of the classical oddball task under water manipulation and verified that the task was effectively implemented. Then, we observed that the removal of bacteria can result in a significantly greater P3 at Fz, FCz, Cz, and FC2 in response to only oddball stimulus when compared with under the water and glycerol manipulations. By using machine learning methods, we demonstrated that the EEG features after different experimental manipulations can be separated with a good accuracy (> 88%).

### Implementation of a classical oddball task

Classical auditory oddball tasks have been used to assess subjects’ attention and cognition^32,33^. In this study, we applied water to the skin on the forehead before the task because it reflects skin’s natural microbial growth. In line with previous reports, we obtained significantly higher P3 amplitudes in the central-parietal area (such as Fz, FCz, Cz, and Pz) for the oddball than for standard trials in the water experiment^33–36^. Hence, the oddball results of our study confirm the effective implementation of the paradigm for assessing attention.

### Skin microbiome altered the brain activities of P3

In this study, we observed that the removal of bacteria from the skin on the forehead in healthy subjects can significantly alter the amplitudes of N2 and P3 during the classical oddball task. The fronto-central P3 peak was thought to positively associate with the attention amount toward the target^37^ and frontal P3 may also reflect the evaluation of response conflict^38^. In this study, we found that after removing bacteria under the alcohol trial, the frontal P3 peaks, but not latency, were significantly increased compared with water and glycerol conditions. Because the used task does not engage any response conflict, the enhanced P3 may suggest that the removal of bacteria enhanced the subject’s attention. However, there was not a significant P3 difference between water and glycerol conditions, though the bacteria populations between the two conditions were significantly different. At first glance, this result seems to contradict our hypothesis that the effect of bacteria on P3 is proportional to the number of bacteria. Upon reflection, there is another possibility: the increased bacteria population did not degrade the brain signals. This is based on two reasons: First, glycerol is not beneficial to all bacteria. Previous studies did demonstrate that applying glycerol can suppress the growth of pathological bacteria and mitigate the inflammation caused^39,40^. Therefore, it is reasonable to assume that glycerol may only promote the growth of certain but not all bacteria in this study. Second, not all bacteria can affect brain signals. This is similar to the idea that “not all bacteria are probiotics.” Indeed, the functional roles of a bacterium depend on the surrounding environment and conditions. For instance, we previously reported that the metabolites of Cutibacterium acnes when given Pluronic F68 can ameliorate ultraviolet B‑induced melanin synthesis and prove to be an effective solution for hyperpigmentation^20^ despite Cutibacterium acnes often being linked to the skin condition of acne^41^.Therefore, we speculate that our insignificant difference in P3 between water and glycerol may be due to the prebiotic effects of glycerol in the sense that it promotes probiotic growth to prevent ‘bad’ bacteria from growing and reducing the brain signal. Finally, it could be that the differences of P3 solely resulted from the arousal effects of alcohol as there is no difference in P3 amplitudes between water and glycerol conditions. If this were true, both the standard and oddball trials after alcohol should exhibit an elevated P3 when compared with that after the water and glycerol manipulations. However, this was not found in the statistical results. Furthermore, it is speculated that the effect of alcohol may be selective, influencing solely the responses to the oddball tones. While we lack direct evidence to rule out the selective effect, we assess this likelihood as relatively low. This is based on the fact that employing alcohol to cleanse the scalp before measurements is a standard procedure in EEG acquisition, yet we did not observe any selective effects during the water or glycerol manipulations. Additionally, it was reported that olfactory stimulation can result in a higher estimated brain source strength during word processing when compared to the control condition where no odor was presented. This effect was observed following a 5-min break^42^. In other words, the neurological effects of odor stimulation fade within 5 min of removing the fragrance stimulation. This is also why we waited 5 min after applying alcohol, to avoid the bias of olfactory stimulation. Further study utilizing odorless solutions can help elucidate the findings.

Regarding the underlying mechanism of the divergence in the P3 amplitudes, one possible molecular target may be the SCFAs' concentration change due to the skin microbiome imbalance (dysbiosis). A previous study found that dysbiosis in the gut (overgrowth or imbalance of microorganisms) could impair cognitive functions^43^. Specifically, gut SCFAs have a well-known effect on the nervous system. The administration of sodium butyrate, a kind of SCFA, elicited improvement in abnormal motor activity and dopamine inadequacy in the mice model of Parkinson disease^44,45^. In human study, PD patients have higher plasma SCFAs (propionic acid, butyric acid, and valeric acid) than faecal SCFAs, such as acetic acid, propionic acid, and butyrate^46^. Wang et al., revealed that children with ASD had a higher concentration of SCFAs than the healthy control^35,47^. In our study, we found that skin samples turned yellow from red phenol after incubation following glycerol and water manipulations, indicating higher acidity in the medium. Because the major metabolic product of the microbiota were SCFAs due to skin bacterial fermentation, the color changes point toward the fact that bacterial fermentation decreases hydrogen (pH) levels of the medium^40,48,49^. Moreover, the levels of acidity were proportional to the amount of bacteria as measured with OD values. Taken together, we interpreted our P3 results as skin microbiome overgrowth in water and glycerol manipulations, leading to an imbalance of SCFAs concentration on the skin. Consequently, P3 amplitudes are reduced in both water and glycerol manipulations while the alcohol manipulation has less microbiome, resulting in a non-decreased P3. Nevertheless, further experiment is needed to identify probiotics that can potentially enhance P3 and the underlying mechanism of this phenomenon.

### ML classifiers to verify the effect of manipulations on EEG activity

To further investigate the impact of bacterial growth on EEG signals, we applied several ML classifiers to separate datasets given the experimental manipulations. As EEG data are complex, many studies have confirmed that ML methods can be used to analyze EEG signals in a multivariate fashion for assisting in the diagnosis of brain disorders^50–52^. In this study, we employed ML methods to separate the datasets of each manipulation with an accuracy greater than 88%, confirming the alternating effects of experimental manipulations on brain signals, despite the paired *t*-test being unable to find a significant difference between glycerol and water. One possible reason behind this divergence may be that the ML methods used a multivariate approach, while the paired-*t*-test utilized a univariate approach^53,54^. Therefore, ML classifiers are more capable of probing the differences between different manipulations.

### Study considerations and future implications

As a pioneering study investigating the relationship between skin microbiota and brain signals, this study was carefully designed, but it still suffers from several shortcomings. First, non-specific bacteria were identified. In this study, we only probed the relation between skin bacteria and EEG changes but did not identify any probiotics. Given that the skin microbiome is a highly diverse and dynamic ecosystem, the composition of skin microbiome can be influenced by various external factors such as environmental conditions, hygiene practices, and individual differences. Therefore, the findings presented in this study should be interpreted with consideration of these potential conditions. For the purpose of further applications, it is critical to identify the probiotics and/or prebiotics that can enhance the effects. The second shortcoming of the study is its relatively small sample size. Given that the between-subject variability in EEG signals is huge, the interpretation of the results is always conditional upon the small dataset. We consider this a pilot study to encourage further investigation into validating the generalization of our results. Thirdly, the classification results may be biased due to model overfitting. For multivariate analysis, we used both significant and non-significant ERP parameters of data features for training. Considering the feature number of 16 and sample size of 20, it is possible to obtain a classification accuracy from an over-fitted predictive model. Further study using adequate samples is needed to verify the results.

Nevertheless, no study has ever attempted to investigate the influence of skin microbiomes on cognitive mechanisms. Understanding the intricate connections between the skin microbiome and cognitive functions may have significant clinical implications. It could pave the way for novel diagnostic tools or therapeutic interventions targeting the microbiome to positively influence cognitive health, potentially opening new avenues in neurology and psychiatry. This pilot study explores the possibility of a skin-brain axis and hope that the findings can help researchers understand the association between skin microbiota and brain functions in the future.

## Conclusion

This study examines whether the change in skin microbiome influences cognitive function of attention as measured with EEG in healthy participants during an oddball task. The results revealed that the removal of the skin bacterial population significantly enhanced the P3 amplitudes along mid-line channels. Machine learning classifiers can separate each manipulation by utilizing EEG data with more than 88% accuracy. To our knowledge, this is the first study that examined the effect of skin microbiome on cognitive function. We hope our findings will motivate further study to explore the underlying mechanism and contribute to further understanding the pathway of the skin-brain axis. Ultimately, understanding the relationship between skin microbiota and brain functions can contribute to the treatment and intervention of diseases that link with this pathway.

## Data Availability

The datasets generated and/or analysed during the current study are not publicly available due to IRB restriction but are available from the corresponding author on reasonable request.
